# Analysis of repeated leukocyte DNA methylation assessments reveals persistent epigenetic alterations after an incident myocardial infarction

**DOI:** 10.1186/s13148-018-0588-7

**Published:** 2018-12-27

**Authors:** Cavin K. Ward-Caviness, Golareh Agha, Brian H. Chen, Liliane Pfeiffer, Rory Wilson, Petra Wolf, Christian Gieger, Joel Schwartz, Pantel S. Vokonas, Lifang Hou, Allan C. Just, Stefania Bandinelli, Dena G. Hernandez, Andrew B. Singleton, Holger Prokisch, Thomas Meitinger, Gabi Kastenmüller, Luigi Ferrucci, Andrea A. Baccarelli, Melanie Waldenberger, Annette Peters

**Affiliations:** 1 Institute of Epidemiology II, Helmholtz Zentrum München, Ingolstädter Landstraβe 1, 85764 Neuherberg, Germany; 2 Mailman School of Public Health, Columbia University, 722 W 168th St, New York, NY 10032 USA; 3 Longitudinal Studies Section, Translational Gerontology Branch, Intramural Research Program, National Institute on Aging, National Institutes of Health, Baltimore, MD 21224 USA; 4 Research Unit Molecular Epidemiology, Helmholtz Zentrum München, Ingolstädter Landstraβe 1, 85764 Neuherberg, Germany; 5 Institute of Human Genetics, Helmholtz Zentrum München, Ingolstädter Landstraβe 1, 85764 Neuherberg, Germany; 6 Department of Epidemiology, Harvard T.H. Chan School of Public Health, 677 Huntington Ave, Boston, MA 02115 USA; 7 VA Normative Aging Study, Veterans Affairs Boston Healthcare System and the Department of Medicine, Boston University School of Medicine, Boston, MA USA; 8 Department of Medicine, Boston University School of Medicine, 72 E Concord St, Boston, MA 02118 USA; 9 Department of Preventive Medicine, Feinberg School of Medicine, Northwestern University, 680 N Lake Shore Dr, Chicago, IL 60611 USA; 10 Robert H. Lurie Comprehensive Cancer Center, Feinberg School of Medicine, Northwestern University, 675 N. St. Clair, Chicago, IL 60611 USA; 11 Department of Preventive Medicine, Icahn School of Medicine at Mount Sinai, 1468 Madison Ave, New York, NY 10029 USA; 12 Geriatric Rehabilitation Unit, Azienda Sanitaria Firenze, Via del Cassero 19, San Casciano in Val di pesa, 50026 Florence, Italy; 13 Laboratory of Neurogenetics, Intramural Research Program, National Institute on Aging, National Institutes of Health, Bethesda, MD 20814 USA; 14 Institut für Humangenetik, Technische Universität München, Arcistrasse 12, 80333 Munich, Germany; 15 DZHK (German Center for Cardiovascular Disease), partner site Munich Heart Alliance, Munich, Germany; 16 Institute of Bioinformatics and Systems Biology, Helmholtz Zentrum München, Ingolstädter Landstraβe 1, 85764 Neuherberg, Germany

**Keywords:** Myocardial infarction, DNA methylation, Epigenetics, Fingerprint, Epigenetic fingerprint, Metabolites, Branched chain amino acid metabolism, Systems biology

## Abstract

**Background:**

Most research into myocardial infarctions (MIs) have focused on preventative efforts. For survivors, the occurrence of an MI represents a major clinical event that can have long-lasting consequences. There has been little to no research into the molecular changes that can occur as a result of an incident MI. Here, we use three cohorts to identify epigenetic changes that are indicative of an incident MI and their association with gene expression and metabolomics.

**Results:**

Using paired samples from the KORA cohort, we screened for DNA methylation loci (CpGs) whose change in methylation is potentially indicative of the occurrence of an incident MI between the baseline and follow-up exams. We used paired samples from the NAS cohort to identify 11 CpGs which were predictive in an independent cohort. After removing two CpGs associated with medication usage, we were left with an “epigenetic fingerprint” of MI composed of nine CpGs. We tested this fingerprint in the InCHIANTI cohort where it moderately discriminated incident MI occurrence (AUC = 0.61, *P* = 6.5 × 10^−3^). Returning to KORA, we associated the epigenetic fingerprint loci with cis-gene expression and integrated it into a gene expression-metabolomic network, which revealed links between the epigenetic fingerprint CpGs and branched chain amino acid (BCAA) metabolism.

**Conclusions:**

There are significant changes in DNA methylation after an incident MI. Nine of these CpGs show consistent changes in multiple cohorts, significantly discriminate MI in independent cohorts, and were independent of medication usage. Integration with gene expression and metabolomics data indicates a link between MI-associated epigenetic changes and BCAA metabolism.

**Electronic supplementary material:**

The online version of this article (10.1186/s13148-018-0588-7) contains supplementary material, which is available to authorized users.

## Background

A myocardial infarction (MI) is characterized by the rupture of a vulnerable plaque into the interior of a coronary vessel resulting in a clotting cascade that obstructs blood flow [1, 2]. Even in the modern era, approximately 5% of MI survivors will experience a recurrent MI within 5 years [3]. For MI survivors, the physiological effects of a MI are widespread and persistent and include anatomical alterations such as ventricular remodeling [4–6]. Additionally, rates of impaired glucose tolerance and diabetes may be high among MI survivors [7, 8], even those without diabetes prior to the MI [7]. Post-MI diabetes is associated with elevated rates of MI recurrence, stroke, and death [8]. Thus, understanding MI-induced molecular alterations and their potential impact on metabolism and vascular physiology may help reduce post-MI co-morbidities and lower event rates. Epigenetics, particularly DNA methylation, is a promising source of molecular data for understanding MI-induced molecular changes.

DNA methylation is the most commonly studied epigenetic mark and is typically an assessment of the frequency with which a methyl group is added to the cytosine in a cytosine-phosphate-guanine (CpG) dinucleotide. This methyl addition at a CpG locus is a stable, yet modifiable, alteration to DNA with direct implications for gene expression and regulation [9]. Although methylation can occur at other dinucleotides, this is mostly restricted to pluripotent cells [10]. DNA methylation at CpG dinucleotides has been associated with MI [11–13], and methylation differences have been observed in healthy versus atherosclerotic tissue from the same individual [14]. Yet, little research has characterized the methylation differences that can occur after an incident MI, and their downstream implications. Here, we used paired samples from the Cooperative Health Research in the Region of Augsburg (KORA) cohort to first identify epigenetic loci that showed methylation changes when comparing pre- and post-MI epigenetic profiles. From these loci, we used KORA and an independent cohort to develop and an “epigenetic fingerprint” of MI, which comprised those loci whose change in methylation is indicative of an incident MI. This epigenetic fingerprint was then evaluated in a third cohort of individuals not used for its development. Finally, we used gene expression and metabolomics data collected in KORA to understand the impact of the epigenetic fingerprint loci on cis-gene expression and peripheral blood metabolites.

## Results

Table 1 contains the clinical covariates for all participating cohorts. To develop the epigenetic fingerprint, we only used loci with methylation data available at both samples for all individuals after all quality control procedures. This high stringency substantially reduced the number of CpGs from the Illumina Infinium Human Methylation 450 K BeadChip array to 24,057 CpGs available for analysis. Of these, 435 CpGs had an FDR *P* < 0.15 in the initial discovery analysis which was designed to be an inclusive analysis to avoid screening out potentially predictive CpGs (*N* = 435, Additional file 1: Table S1). We retained 174 of these 435 CpGs which has non-zero betas in the elastic net model run on KORA data (Additional file 1: Table S2). To further refine the predictive model, we used a second elastic net model in Normative Aging Study (NAS), and of the 174 CpGs from KORA, retained those 11 CpGs with non-zero betas in the NAS (Additional file 1: Table S3). The difference in methylation between baseline and follow-up for these 11 CpGs significantly discriminated (*P* < 0.05) occurrence of MI in KORA, NAS, and Invecchiare nel Chianti (InCHIANTI), an independent cohort not used to select the CpGs (Additional file 1: Table S4).

**Table 1:** Clinical covariates for KORA, NAS, and InCHIANTI

a. Clinical covariates at baseline and follow-up exams						
	KORA		NAS		InCHIANTI	
	Baseline	Follow-up	Baseline	Follow-up	Baseline	Follow-up
*N*	1103	1103	344	344	443	443
*N* MI	–	13	–	14	–	50
Time to MI—years; mean (SD)	–	4.08 (2.2)	–	2.76 (1.3)	–	6.47 (2.9)
FU time—years; mean (SD)	–	7.10 (0.22)	–	8.52 (3.2)	–	9.12 (0.21)
Age—years; mean (SD)	54.5 (8.87)	61.5 (8.9)	70.9 (6.6)	74.5 (6.6)	61.7 (16.2)	70.8 (16.2)
Males—*N* (%)	558 (50.6%)	558 (50.6%)	100%	100%	206 (46.5%)	206 (46.5%)
BMI—kg/m^2^; mean (SD)	27.7 (4.5)	28.1 (4.8)	28.1 (4.1)	27.7 (4.1)	27.0 (3.9)	27.0 (4.3)
Current smokers—*N* (%)	212 (19.2%)	161 (14.6%)	17 (4.94%)	16 (4.65%)	89 (20.1%)	45 (10.2%)
Former smokers—*N* (%)	417 (37.8%)	469 (42.5%)	220 (64.0%)	222 (64.5%)	105 (23.7%)	151 (34.1%)
LDL-C—mg/dL; mean (SD)	144 (40.1)	140 (35.8)	–	–	135 (35.9)	124 (31.8)
HDL-C—mg/dL; mean (SD)	57.5 (16.9)	56.2 (14.8)	51.3 (14.1)	50.6 (13.5)	56.1 (14.2)	56.5 (15.2)
Hypertension—*N* (%)	484 (44.2%)	522 (47.4%)	209 (60.8%)	232 (67.4%)	221 (49.9%)	247 (55.8%)
Type 2 diabetes—*N* (%)	39 (3.54%)	105 (9.53%)	36 (10.5%)	48 (14.0%)	38 (8.6%)	49 (11.1%)
b. Clinical covariates at baseline for individuals who did not develop an MI (controls) vs those who did (cases)						
	KORA		NAS		InCHIANTI	
	Controls	Cases	Controls	Cases	Controls	Cases
*N*	1090	13	330	14	393	50
Age—years; mean (SD)	54.4 (8.9)	58.9 (7.2)	70.9 (6.5)	69.3 (8.4)	60.9 (16.7)	67.8 (9.4)
Males—*N* (%)	548 (50.3%)	10 (76.9%)	100%	100%	181 (46.1%)	25 (50.0%)
BMI—kg/m^2^; mean (SD)	27.7 (4.4)	31 (7.1)	28.0 (4.1)	29.0 (4.0)	26.9 (3.9)	27.3 (3.46)
Current smokers—*N* (%)	208 (19.1%)	4 (30.8%)	16 (4.84%)	1 (7.14%)	80 (20.4%)	9 (18.0%)
Former smokers—*N* (%)	412 (37.8%)	5 (38.5%)	210 (63.6%)	10 (71.4%)	88 (22.4%)	17 (34.0%)
LDL—mg/dL; mean (SD)	144 (40.2)	144 (36.7)	–	–	134 (35.9)	144 (34.6)
HDL—mg/dL; mean (SD)	57.7 (16.8)	42 (11.7)	51.7 (14.1)	41.7 (8.5)	56.0 (14.3)	57.6 (13.2)
Hypertension—*N* (%)	476 (44%)	8 (61.5%)	201 (60.9%)	8 (57.1%)	187 (47.6%)	34 (68.0%)
Type 2 diabetes—*N* (%)	37 (3.4%)	2 (15.4%)	32 (9.70%)	4 (28.6%)	31 (7.9%)	7 (14.0%)
c. Clinical covariates at follow-up for individuals who did not develop an MI (controls) vs those who did (cases)						
	KORA		NAS		InCHIANTI	
	Controls	Cases	Controls	Cases	Controls	Cases
*N*	1090	13	330	14	393	50
Time to MI—years; mean (SD)	–	4.08 (2.2)	–	2.76 (1.3)	–	6.47 (2.9)
FU time—years; mean (SD)	7.1 (0.22)	7.2 (0.38)	8.8 (2.97)	2.8 (1.3)	9.1 (0.21)	9.1 (0.21)
Age—years; mean (SD)	61.4 (8.9)	65.9 (7.2)	74.5 (6.5)	73.3 (8.2)	70.0 (16.7)	76.9 (9.5)
Males—*N* (%)	548 (50.3%)	10 (76.9%)	100%	100%	181 (46.1%)	25 (50.0%)
BMI—kg/m^2^; mean (SD)	28.1 (4.71)	32.6 (7.24)	27.7 (4.1)	28.3 (4.2)	27.0 (4.35)	27.0 (3.72)
Current smokers—*N* (%)	159 (14.6%)	2 (15.4%)	15 (4.6%)	1 (7.1%)	42 (10.7%)	3 (6.0%)
Former smokers—*N* (%)	462 (42.4%)	7 (53.8%)	212 (64.2%)	10 (71.4%)	128 (32.6%)	23 (46.0%)
LDL-C—mg/dL; mean (SD)	140 (35.8)	111 (16.8)	–	–	123 (31.9)	127 (31.4)
HDL-C—mg/dL; mean (SD)	56.4 (14.8)	41.5 (10.2)	51.1 (13.6)	40.3 (6.0)	56.4 (15.3)	57.3 (14.6)
Hypertension—*N* (%)	513 (47.2%)	9 (69.2%)	219 (66.3%)	13 (92.9%)	216 (55.0%)	31 (62.0%)
Type 2 diabetes—*N* (%)	98 (9.0%)	7 (53.8%)	43 (13.0%)	5 (35.7%)	44 (11.2)	5 (10.0%)

Summary given as mean (SD) for continuous variables: time to myocardial infarction (time to MI), follow-up time (FU time), age, body mass index (BMI), low-density lipoprotein cholesterol (LDL-C), and high-density lipoprotein cholesterol (HDL-C). Summary given as the number and percentage responding positively for binary variables: males, current smokers, former smokers, hypertension, and type 2 diabetes

**Fig. 1:**
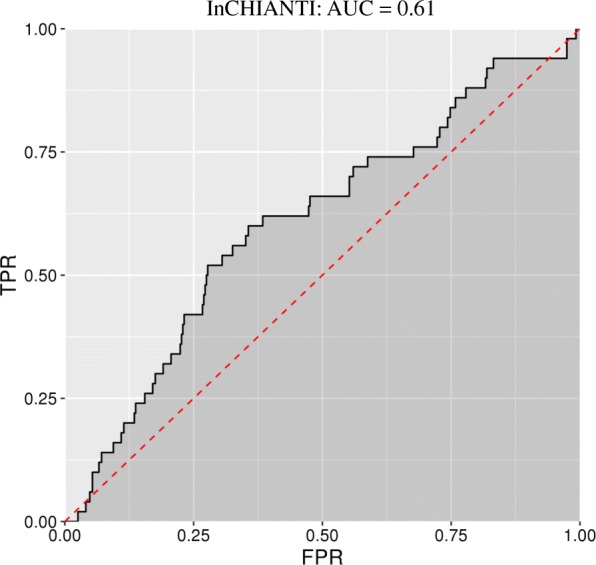
Receiver operating characteristic (ROC) curves for the epigenetic fingerprint. The ROC curve for InCHIANTI for the epigenetic fingerprint. The fingerprint was developed in KORA and NAS and independently evaluated (replicated) in InCHIANTI where is significantly discriminated MI occurrence (*P* = 6.5x10^-3^). *AUC* area under the curve; *FPR* false positive rate; *TPR* true positive rate

Usage of medication is commonly prescribed after an MI closely correlated with the occurrence of an incident MI (Additional file 1: Table S5). Using KORA F4, we tested for associations between these 11 CpGs and the usage of seven classes of medications commonly prescribed after a MI. After a correction for the 77 tests performed, two CpGs were associated with medication usage in KORA F4: one with the stoppage of diuretics (cg19569340) and one with the stoppage of anti-platelet medications (cg02628823, Additional file 1: Table S6). Thus, our final epigenetic fingerprint was composed of nine CpGs (Table 2). These nine CpGs significantly discriminated incident MI occurrence in both KORA and NAS, and this discrimination was independently evaluated in InCHIANTI (Fig. 1, Table 3).

**Table 2:** The 9 CpGs that composes the epigenetic fingerprint of MI

CpG	CHR	BP (Mb)	Gene/Locus	Relation to island (UCSC)	Enhancer	DHS
cg23541257	1	53.8	*KCNN1*	South shelf		
cg08193363	1	55.35	*FRY*	North shore		Yes
cg21609024	1	145	*LRP8*			
cg10073091	1	171	*DHCR24*	North shore		
cg07311024	6	166	*GLIPR1L2*	Island		Yes
cg23074119	12	75.79	*ALKBH1*			
cg11955541	13	32.61	*PDE4DIP*	Island	Yes	Yes
cg00699486	14	78.17				
cg03458344	19	18.1	C1orf129			

Annotations to genes are based on physical proximity. Annotations are based on location and provided in the manifest file for the 450 K methylation array by Illumina

*BP* base pair location of the CpG, *CpG* methylation probe, *DHS* DNase I hypersensitivity site, *Mb* megabases, *UCSC* University of California, Santa Cruz genome browser-based annotation

**Table 3:** Model statistics for the epigenetic fingerprint for KORA, NAS, and InCHIANTI

	*N*	*N* MI	AUC	*P* (AUC)
KORA	1103	13	1.00	2.7 × 10^−10^
NAS	344	14	0.94	1.3 × 10^−08^
InCHIANTI	443	50	0.61	6.5 × 10^−03^

The epigenetic fingerprint was developed in KORA and NAS and independently evaluated in InCHIANTI. Area under the curve (AUC) is based on logistic regression models

### Association with gene expression

We associated each of the epigenetic fingerprint CpGs with gene expression in KORA F4 using 713 samples. Given the power to detect associations, we only examined cis-gene expression (1 Mb window) and examined both nominal (*P* < 0.05) associations and those significant after a Bonferroni correction for the number of genes within each 1 Mb window (14–87 genes, Additional file 1: Table S7). In an age, sex, and technical factor-adjusted model, five epigenetic loci were at least nominally associated with cis-gene expression, with cg100703091 significantly associated with gene expression probes for 24-dehydrocholesterol reductase (*DHCR24*) and transcription elongation factor A N-terminal and central domain containing 2 (*TCEANC2*) (Table 4). We examined the Biobank-based Integrative Omics Studies (BIOS) consortium QTL browser for potential replication of the associations (https://genenetwork.nl/biosqtlbrowser/) [15, 16]. BIOS only reported genome-wide significant, independent associations and did not have overlap with our results.

**Table 4:** Integration of epigenetic fingerprint loci with gene expression

Chrom	GEX Probe	CpG	BP	Annotated gene	Beta	SE	P	Probe gene
1	ILMN_1725510	cg10073091*	55.35	*DHCR24*	− 1.76	0.51	0.0006	*DHCR24*
1	ILMN_1673544	cg10073091*	55.35	*DHCR24*	2.37	0.73	0.0012	*TCEANC2*
1	ILMN_1681340	cg10073091	55.35	*DHCR24*	− 4.29	1.54	0.0055	*HSPB11*
19	ILMN_1654571	cg23541257	18.10	*KCNN1*	− 1.52	0.63	0.02	*FCHO1*
19	ILMN_1742917	cg23541257	18.10	*KCNN1*	1.00	0.45	0.03	*NXNL1*
14	ILMN_1758038	cg23074119	78.17	*ALKBH1*	− 25.80	11.89	0.03	*ALKBH1*
1	ILMN_1671568	cg21609024	53.80	*LRP8*	2.02	0.94	0.03	*ECHDC2*
19	ILMN_1797005	cg23541257	18.10	*KCNN1*	− 1.36	0.64	0.03	*PGLS*
6	ILMN_2121272	cg00699486	166.14		− 0.42	0.21	0.04	*PDE10A*

*Beta* effect estimate for gene expression—methylation association, *BP* chromosomal location of the CpG, *P* unadjusted *P* value, *SE* standard error

Of the nine epigenetic fingerprint loci, five were at least nominally associated (*P* < 0.05) with cis-gene expression (1 Mb window). Two, marked with an asterisk (*), were associated after adjusting for the number cis-genes, i.e., genes within 1 Mb. Annotated gene refers to the proximity-based annotation of the methylation probe (CpG) to a gene by Illumina. Probe gene refers to the gene associated with the gene expression (GEX) probe

### Multi-omic pathway visualization

**Fig. 2:**
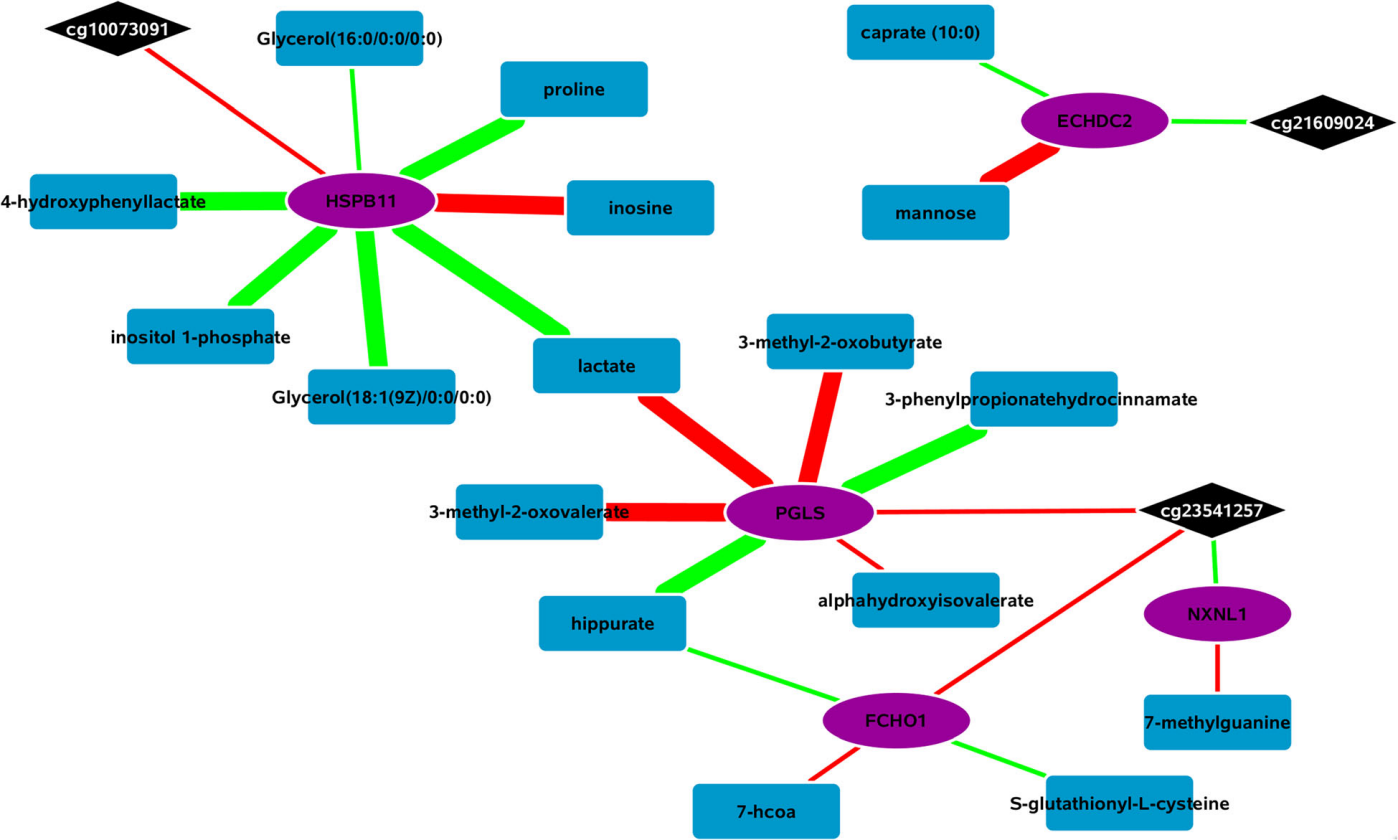
Integration of gene expression and metabolomics networks into the epigenetic fingerprint loci. Black diamonds represent epigenetic loci, blue rectangles represent metabolites, and purple ellipses represent genes. Red edges indicate negative associations/correlations and green positive. Thicker edges represent the 12 FDR significant metabolite–gene expression correlations. For improved visualization all metabolite-gene expression associations with *P* < 0.001 are represented

In order to visualize the metabolic pathways associated with our epigenetic fingerprint, we integrated the CpG-gene expression associations (*P* < 0.05) with a published Spearman correlation-based gene expression-metabolomics network [17]. Accounting for multiple probes per gene, 3000 associations were examined. There were 12 FDR significant associations, two of which were Bonferroni significant after a multiple-testing correction (Fig. 2**,** Additional file 1: Table S8). Three epigenetic fingerprint CpGs accounted for all genes with suggestive metabolite associations. Heat shock protein family B (small) member 11 (*HSPB11*) was the most represented gene in the network, with seven suggestive metabolite associations, six of which were FDR significant.

The majority of the network centered on two “hubs” linked by the metabolite lactate. One hub has *HSBP11* at the center, and the other hub has 6-phosphogluconolactonase (*PGLS*) at its center. The *PLGS* hub has an additional branch that incorporates FCH domain only 1 (*FCHO1*) and nucleoredoxin like 1 (*NXNL1*) while all members of the *HSPB11* hub directly connect to that gene. Besides the “twin-hub” main body of the network, there is one disconnected section representing the cg21609024–enoyl-CoA hydratase domain containing 2 (*ECHDC2*) association. *ECHDC2* had an FDR significant association with mannose and a suggestive association with caparic acid (caparate (10:0)).

## Discussion

Epigenetic changes to DNA have a profound impact on cellular regulation and health. Environmental exposures, such as smoking [18–21] and air pollution [22, 23], may cause changes to the epigenome, and aberrant DNA methylation changes may be a risk factor for outcomes such as obesity [24], diabetes [25, 26], and cardiovascular disease (CVD) [11, 27, 28]. What is less understood is how changes in health status can produce long-term alterations in the epigenetic profile and the subsequent implications for the downstream regulation of gene expression and biochemical pathways. Here, we have shown that the occurrence of an incident MI is associated with a broad array of epigenetic changes and that a subset of the loci, whose change in methylation is associated with incident MI, compose an “epigenetic fingerprint” of MI that generalizes across populations from various geographic regions and ethnicities. The complete map of epigenetic changes as a result of an incident MI is likely to be highly personalized and heterogeneous and will depend on factors such as the timing of the MI (early vs late life), the epigenetic profile of the individual at the time of MI, the risk factors that contributed to the MI, and even the triggering mechanism of the MI. However, we believe the epigenetic loci highlighted by our analyses represent a picture of some of the epigenetic changes that may result from an MI occurrence.

### Epigenetic loci which compose the epigenetic fingerprint

There were nine epigenetic loci which composed the epigenetic fingerprint and provided substantial discrimination in the occurrence of a first MI (Table 2). These nine loci were not associated with MI risk in either of two recent epigenome-wide association studies for MI [11, 13]. The nine CpGs were annotated to eight genes based on their location. Of these eight genes, two are known to be associated with cardiovascular disease: low-density lipoprotein receptor-related protein 8 (*LRP8*) and potassium calcium-activated channel subfamily N member 1 (*KCNN1*). Low-density lipoprotein concentration is a known risk factor for MI with growing evidence for a causal association with vascular disease [29–31]. *LRP8* has been implicated as a diagnostic marker for familial and early-onset CVD [32]. *KCNN1* is a calcium-activated potassium channel expressed in heart and other tissues. Blocking the homolog of this channel in rats can reduce ventricular fibrillation and ventricular tachycardia during induced acute myocardial infarction [33]. *KCNN1* is also involved in the electrical remodeling of the heart during chronic atrial fibrillation [34].

*DHCR24* and alkB homolog 1, histone H2A dioxygenase (*ALKBH1*) were also annotated to epigenetic fingerprint CpGs. *DHCR24* encodes a reductase that catalyzes the final step in cholesterol biosynthesis, the conversion of desmosterol to cholesterol [35], and the mutations in *DHCR24* cause accumulation of desmosterol [36]. Methylation at *DHCR24* is associated with body mass index [24, 37]. Desmosterol accumulation underlies many of the downstream responses to foam cell formation [38], which are causally implicated in MI. *DHCR24* also has roles in response to both acute and chronic oxidative stress [39], and methylation in *DHCR24* is regulated by low-density lipoprotein cholesterol and associated with *DHCR24* gene expression [40]. Mendelian randomization analyses indicate that methylation in *DHCR24* may be causally regulated by low-density lipoprotein cholesterol levels in blood [40]. Thus, it is possible that post-MI methylation changes in *DHCR24* are driven by changes in blood lipids. *ALKBH1* encodes for a response protein to DNA alkylation damage and specifically modifies the methylation and, therefore, regulation of histone H2A [41].

Of the remaining three epigenetic fingerprint CpGs, one was annotated to GLI pathogenesis related 1 like 2 (*GLIPR1L2*), which is associated with immune system cells and macrophage differentiation [42], and is regulated by DNA methylation [43]. One was annotated to phosphodiesterase 4D interacting protein (*PDE4DIP*), which is associated with large vessel stroke [44], and the final CpG was annotated to FRY microtubule binding protein (*FRY*), which is associated with microtubule regulation/spindle formation [45].

### Association of fingerprint loci with gene expression

To understand the transcriptional regulatory potential of our epigenetic fingerprint loci, we associated each CpG loci with the expression of genes within 1 Mb (Table 4). The CpG site cg00699486 did not have a location-based annotation in the Illumina annotation files, but was nominally associated with the expression of phosphodiesterase 10A (*PDE10A*), a gene linked to vascular remodeling [46]. Only cg10073091 (*DHCR24*) and cg23074119 (*ALKBH1*) were associated with the expression of the genes to which they were annotated based on proximity. Cg10073091 was the only locus to be associated with gene expression (*DHCR24* and *TCEANC2*) after a multiple-test correction and was also nominally associated with *TCEANC2* and *HSPB11* expression. Cg1007391 is located in the first exon of *DHCR24* and overlaps with transcription factors linked to the vasculature, inflammation, and hypoxia such as STAT3 and ELF-1 [47–49], which may account for its multiple associations.

### Integration with metabolomics data

To understand the biochemical impact of epigenetic changes associated with incident MI, we linked our epigenetic fingerprint-gene expression associations with a published network of transcriptome-metabolome associations [17]. Three epigenetic fingerprint loci were linked to metabolites via transcriptomic regulation. When considering all suggestive (*P* < 0.001) transcriptome-metabolome associations, these three CpGs linked to two distinct sub-networks. The larger sub-network was composed of two hubs centered on *HSPB11* and *PGLS* with lactate linking the two (Fig. 2). The network hub centered on *PGLS* contained multiple metabolites linked to branched chain amino acid (BCAA) metabolism. BCAAs are associated with obesity [50], insulin resistance [51, 52], diabetes [53], cardiovascular disease [54, 55], and mortality [56]. *PGLS* was also significantly associated with hippurate, a metabolite produced by microbial metabolism in the gut. Hippurate is often used as a marker for renal function and has been associated with diabetes, blood pressure, and atherosclerosis, though these associations are possibly secondary to renal dysfunction [57, 58]. *PGLS* encodes for 6-phosphogluconase, which is differentially expressed in the pancreas of diabetic mice as compared to wildtype mice [59].

*HSBP11* sits at the center of the other network hub and had the most FDR significant associations (6). Proline is associated with immune function in the gut [60] and is metabolized by the gut microbiome to produce ornithine and arginine [60]. Ornithine and arginine are associated with atherosclerosis [61, 62] and CVD [63, 64]. Mutual association with lactate linked the *PGLS* and *HSPB11* hubs, and *HSPB11* was also linked to a BCAA metabolite (4-hydroxyphenyllactate). In a study comparing obese and lean humans, proline, lactate, and BCAAs were all elevated in the obese individuals [50], and, like the BCAAs, lactate is linked to insulin resistance [50–53, 65]. High rates of diabetes and glucose intolerance have been reported amongst MI survivors [7, 8], a trend that has been increasing over time [66]. Even individuals without diagnosed diabetes prior to MI have high rates of impaired glucose tolerance and diabetes after an MI [7]. Thus, the component of the epigenetic fingerprint encompassing cg23541257 and cg10073091, and their downstream transcriptomic and metabolomic associations, may reflect post-MI epigenetic regulation related to obesity, insulin resistance, and diabetes.

Of the remaining metabolites, only mannose had a previously reported association with CVD or MI. *ECHDC2* was significantly associated with mannose, a water-soluble fiber that may have protective effects for MI [67]. *ECHDC2* encodes for a mitochondrial protein involved in mediating susceptibility to myocardial ischemia/reperfusion injury in rats and was shown to increase BCAA metabolism in rats [68].

### Limitations

The primary limitation of this study is the low numbers of incident MI events. With fewer than 20 incident MI events in each KORA and NAS, the epigenetic fingerprint was developed based on limited event observations. In post hoc power calculations for the initial, screening, EWAS highlight this with many observed effects below the traditional 80% power cutoff (Additional file 1: Figure S1). However, our multi-staged design still allowed for the removal of CpGs that do not contribute to prediction, and the epigenetic fingerprint provided significant discrimination in an independent cohort, with a larger number of events than either KORA or the NAS. A related limitation is the varying follow-up times for the studies and event rate for the cohorts. This could introduce variability which might obscure some CpGs that may have improved our MI discrimination models. However, the varying follow-up times helps insure that the CpGs in the final model are those whose post-MI alterations in methylation are stable over varying follow-up time windows. Showing replicability across cohorts with varying incidence rates, which may occur due to sampling decisions or underlying clinical conditions, helps to demonstrate the robustness of the results. Another limitation of this study is that the vast majority of the CpGs from the 450 K array were missing in > 1 individuals in KORA and thus not used. Though this restriction insured only the highest quality CpGs were used, insured all CpGs could participate in the elastic net model, and improved power in the EWAS, it does mean that CpGs that possibly significantly discriminate MI were excluded. Future studies may consider imputation or sequencing methods to improve CpG availability. Another limitation is that we cannot discount the possibility that some of the MI fingerprint discrimination is driven by the effect of lifestyle changes. Though the EWAS did adjust for changes in BMI, physical activity, smoking, and alcohol consumption, it is still possible that unmeasured confounding from lifestyle changes such as diet or occupation remains. Another limitation of this analysis is the generalizability. Both KORA and InCHIANTI contained only European ancestry individuals, and while NAS contained some non-European ancestry individuals, the proportion was extremely small. It would be beneficial for future studies to test these epigenetic loci to see if they change in methylation discriminates between individuals with and without an incident MI in ethnically diverse cohorts. The DNA methylation-gene expression associations observed in KORA were not observed in results published in the BIOS consortium QTL browser (https://genenetwork.nl/biosqtlbrowser/) [15, 16]. The browser only reported the independent results with FDR corrected *P* < 0.05; thus, our results may simply not have achieved this stringent statistical cutoff. Additionally, epigenetic differences between BIOS and KORA, due to lifestyle or exposure history, may account for the lack of overlap between the results. Leukocyte DNA methylation may not reflect methylation with cardiac tissue; however, it may be a proxy for methylation there or reflect systemic changes indicative of a recent MI. Additionally, because the elastic net model prunes sets of highly correlated predictors at random, the selected loci may only be proxies for those whose change is truly due to the occurrence of an incident MI. Finally, with only one time point after the occurrence of the incident MI, we are unable to determine the rate of change of the epigenetic fingerprint loci after an MI. Whether these epigenetic changes occur immediately and then remain stable or occur slowly and continue to diverge after an incident MI is a topic for future investigations in cohorts with three or more methylation assessments.

## Conclusion

Incident MI is significantly associated with changes in methylation at multiple CpGs, nine of which compose an epigenetic fingerprint representing stable, medication-independent, MI-associated alterations in the epigenetic profile. The loci that composed the epigenetic fingerprint were associated with the expression of genes related to cholesterol metabolism (*DCHR24*) and vascular remodeling (*PDE10A*). By integrating the epigenetic-transcriptomic associations with metabolomics data, we were able to visualize an integrated ‘omics network revealing the potential biochemical effects of these epigenetic loci. Three of the nine loci were incorporated into this integrated ‘omics network, each of which linked to a gene or metabolite implicated in BCAA metabolism. The most connected genes, *PLGS* and *HSPB11*, were also linked to gut microbiome associated metabolites. Taken together, this multi-omic network suggests that epigenetic changes after an incident MI may be linked to BCAA metabolism and possibly inform on the development impaired glucose tolerance and diabetes secondary to MI.

## Methods

### Study cohorts

To develop the epigenetic fingerprint, we used paired samples from the KORA S4 and KORA F4 cohorts. KORA S4 is the baseline examination of individuals living in the Augsburg region, Germany, with examinations conducted from 1999 to 2001 [69]. KORA F4 is a follow-up survey of KORA S4 and was conducted from 2006 to 2008 [70]. After removing individuals with previous MI at the KORA S4 enrollment, there were 1103 individuals with paired methylation data from KORA S4 (i.e., “baseline”) and KORA F4 (i.e., “follow-up”). Methylation at both time points was assessed via the Illumina Infinium HumanMethylation450k platform. MI events were assessed for all KORA participants via their enrollment in the Augsburg MI registry [71]. There were 13 incident MI occurrences between baseline and follow-up.

We refined the epigenetic fingerprint in the Normative Aging Study (NAS) [72] and replication was performed in the Invecchiare nel Chianti (InCHIANTI) cohort [73]. NAS is an ongoing longitudinal study established in 1963. Men free of any known chronic disease or medical conditions and aged 21–80 were recruited at baseline and followed-up with medical examinations every 3–5 years. Examinations included medical and lifestyle questionnaires as well as physical exams and blood collection for laboratory tests. Methylation was assessed using the Illumina Infinium HumanMethylation450k platform. There were 344 NAS participants, 333 of which were of European ancestry. We observed 14 incident MI events in NAS, with all but one occurring in European ancestry individuals.

InCHIANTI is a population-based cohort selected from residents of the Chianti region of Tuscany, Italy. Individuals aged 20 and older were recruited and assessed at both a baseline (1998–2000) and 9-year follow-up (2007–2009) examination. As in KORA and NAS, methylation was assessed using the Illumina Infinium HumanMethylation450k platform. MI was assessed using questionnaires at both baseline and follow-up examinations, and events adjudicated using data from hospital records, electrocardiogram diagnostics, and cardiac enzyme assays. Individuals with prevalent MI at baseline were excluded. This left 443 InCHIANTI participants for analysis; 50 had an incident MI during follow-up.

Only incident MIs that occurred between the baseline and follow-up examinations were treated as events. All studies received written and informed consent from all participants at all time points and were approved by their respective ethical boards. Complete descriptions of all studies appear in the Additional file 1, and clinical covariates for all cohorts can be found in Table 1.

### Normalization and technical factors for methylation data

In KORA, the methylation beta values were normalized using a beta-mixture inter-quartile (BMIQ) normalization [74] after background correction. To adjust for technical factors, 20 principal components derived from the control probes on each chip were used [75]. Estimated cell counts [76] were also included in the analysis to adjust for heterogeneity in cell composition. This normalization, technical factor adjustment, and inclusion of the estimated cell counts match previous analyses of KORA methylation data [24, 77].

NAS also used BMIQ normalization after background correction to normalize the methylation probe values. To adjust for technical factors, variables for the plate and position of the chip, row, and column were included in each analysis as well as estimated cell counts [76]. InCHIANTI used the background correction and dye-bias equalization method as implemented in *noob* in the *minfi* R package [78, 79], and probes were normalized using the approach implemented in *dasen* in the *wateRmelon* R package [80]. Technical factors adjusted for in the InCHIANTI models were estimated cell counts [76], batch, slide, and array. Full details on the design, methylation assessment and normalization, and technical factor adjustment can be found in the Additional file 1.

### Development of the epigenetic fingerprint

We developed the epigenetic fingerprint via a multi-step process in the KORA and NAS cohorts. In the first step, we used an epigenome-wide association study (EWAS) in KORA to determine CpGs whose change in methylation from baseline to follow-up is associated with an incident MI occurring during this time interval. We used a generalized estimating equation (GEE) model to estimate the degree to which change in methylation (from a defined baseline of 0 to the observed difference) is influenced by the occurrence of an incident MI between baseline and follow-up while accounting for covariates at both baseline and follow-up and for the within individual correlation between covariates. The use of the GEE model in this scenario could shrink the standard errors, which would inflate the number of false positives. However, this is explicitly accounted for in the multi-stage design by which we allow for false positives in the first stage to maximize inclusion of potentially predictive CpGs. False positives are removed by the use of penalized regression, elastic net model, in two independent cohorts before the final model is determined. False positive CpGs which are not predictive of the outcome would be eliminated by the elastic net when applied to an independent cohort not used in the CpG discovery. Prior to calculating the change in methylation over time, the methylation β values were adjusted for cohort specific technical factors including estimated cell counts [76]. The residuals from this regression were used to estimate the follow-up—baseline methylation difference (ΔCpG). At baseline, ΔCpG was defined as 0 (Additional file 1).

We associated ΔCpG in KORA with a binary indicator for the occurrence of an incident MI between the two samplings using a single adjustment model which adjusted for age, sex, body mass index (BMI), type 2 diabetes, hypertension, physical activity, pack-years of smoking, and alcohol consumption (g/day) both at baseline and follow-up. Physical activity was a binary variable indicating if the individual considered themselves to be active or not. Pack-years of smoking was defined as the packs of cigarettes smoked per day (1 pack = 20 cigarettes) times the years spent smoking. In order to be able to calculate the methylation difference for each individual and each methylation locus, we restricted the EWAS to those CpGs with no missing values (*N* = 24,057). This allowed any CpGs identified in the EWAS to be carried forward into the elastic net model, which does not allow for missing values in the predictors. We created the epigenetic fingerprint from those CpGs with a false discovery rate (FDR) [81] *P* < 0.15. We used a liberal FDR cutoff at this stage to maximize inclusion of CpGs that showed even a weakly suggestive level of association, while acknowledging the potential inclusion of many “false positive” CpGs which were to be eliminated using an elastic net model in the next stage of analysis. We used the longpower package in R to conduct a post hoc power analysis which accounted for the disparity in observed MI events versus total samples as well as the correlation of the CpGs between examinations and among those who had an MI versus those who did not (Additional file 1**:** Figure S1). The expectation of low power in this initial, screening, EWAS is a primary motivation for the use of a penalized regression method (elastic net) in an independent sample to down weight predictors that initially arose due to noise and low power. Those CpGs that did not contribute to the fingerprint discrimination would be excluded in the elastic net model.

We used an elastic net model (logit link) implemented in KORA for the initial selection of the epigenetic fingerprint CpGs from those with FDR *P* < 0.15 in the EWAS. Given that the model would be overfit, we refined the elastic net using independent samples from NAS, by estimating a second elastic net model using only those CpGs with non-zero coefficients from the initial KORA elastic net model. We assessed the predictive power of these models via the area under the receiver operating characteristic curve (AUC) [82, 83]. As these epigenetic loci could be associated with post-MI medication usage [84, 85], we used KORA to associate each medication type commonly prescribed post-MI with the CpGs retained in the NAS elastic net model. The CpGs in the model had already been assessed for associates with MI independent of age, body mass index, smoking, alcohol consumption, type 2 diabetes, and hypertension in the EWAS; thus, those confounders were not retested here. We used ΔCpG as our outcome and tested each locus in the epigenetic fingerprint for associations with the use of seven commonly prescribed medication types: beta-blockers, ACE-inhibitors, diuretics, angiotensin receptor blockers, calcium channel blockers, statins, and anti-platelet medications [86, 87]. We encoded each medication as a factor variable and tested for epigenetic differences associated with either stopping or starting the medication. A Bonferroni correction was used to determine medication-associated CpGs, and any associated CpGs were removed from the CpGs that composed the epigenetic fingerprint. We used a Bonferroni correction to reduce the probability of removing epigenetic loci not truly associated with medication usage.

Thus, the final epigenetic fingerprint loci were composed of those CpGs retained in NAS but not associated with medication usage. The discrimination of this final list of CpGs was retested via logistic regression in KORA and NAS, to allow for reweighting of the coefficients after removing medication-associated CpGs. The independent out-of-sample discrimination of the epigenetic fingerprint was evaluated, using logistic regression, in InCHIANTI.

All analyses were performed in R [88]. Generalized estimating equations used in the EWAS were implemented in *geepack* [89], and the elastic net models used were implemented in *glmnet* [90]. The packages *ROCR* [91] and *verification* were used for plotting and calculating statistics associated with receiver operating characteristic (ROC) curves.

### Association of epigenetic fingerprint with gene expression and integration with metabolomics networks

As regulation of gene expression is a primary consequence of DNA methylation, we associated each of the CpGs that composed the epigenetic fingerprint with gene expression in KORA F4. Gene expression was assessed on the Illumina HumanHT-12v3 array. A total of 713 individuals had both methylation and gene expression in KORA F4. We associated each epigenetic fingerprint CpG with the gene expression of all genes within 1 Mb of the loci (cis-genes). We adjusted for age and sex in the models as well as estimated cell counts and technical factors. As a multiple-testing correction, we used a Bonferroni correction for the number of genes within the 1 Mb window for each CpG.

**Fig. 3:**
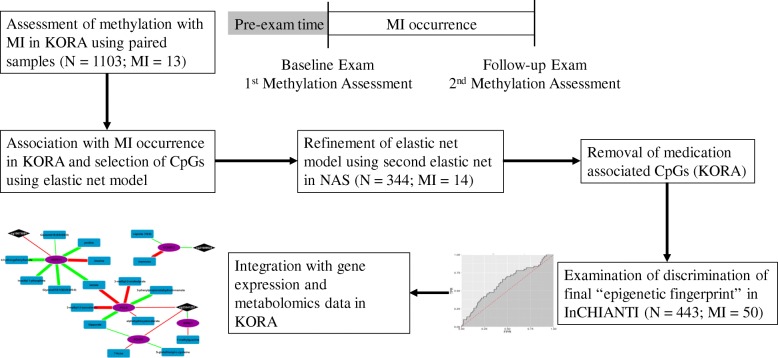
Development of the epigenetic fingerprint. Flowchart depicting the discovery and validation of CpGs where the change in methylation between baseline and follow-up exams is associated with the occurrence of a MI, i.e., an epigenetic fingerprint of MI. In the final step, the CpGs which compose the epigenetic fingerprint are integrated into a gene expression and metabolomics network to better understand their functional impact

We created a multi-omics network model by integrating the CpG-gene expression associations with integrated metabolomics data using gene expression-metabolomics associations based on KORA F4 data. The KORA F4 data came from a published Spearman correlation-based blood metabolome-transcriptome network [17]. We extracted the relevant gene expression probes and metabolites from this network, removing any metabolites of unknown structure. Based on the reported Spearman correlation, we calculated the *P* values and used an FDR correction to determine significant associations. Finally, we used Cytoscape® [92] to integrate all suggestive (*P* < 0.001) gene expression-metabolomics associations with any nominal (*P* < 0.05) methylation-gene expression associations. Both *P* value cutoffs were selected to allow for better visualization of the implicated pathways with a more stringent cutoff used for the gene expression-metabolomics data to account for the larger number of tests. A diagram of the procedure for the development of the epigenetic fingerprint and integration with gene expression and metabolomics data is given in Fig. 3.

## Additional file

Additional file 1: Supplemental methods, **Table S1.** CpGs from KORA EWAS. Those CpGs with a false discovery rate *P* < 0.15 in an epigenome-wide association study in KORA using the difference in methylation between the baseline and follow-up exams as the outcome (after adjustment for technical factors) and the occurrence of an MI as the predictor while adjusting for clinical covariates (at both baseline and follow-up) in a generalized estimating equations model. **Table S2.** The 174 CpGs which were retained from the initial elastic net model performed in KORA. **Table S3.** Epigenetic loci with non-zero coefficients from the NAS elastic net model. **Table S4.** AUC for the model fit with the loci with non-zero betas from the NAS elastic net in KORA, NAS, and InCHIANTI. **Table S5.** Medication usage in KORA at baseline and follow-up. We divide out the medication usage in KORA at (a) baseline and (b) follow-up for those individuals who did not develop and incident MI during the observation time (MI free) vs those that did (MI cases). **Table S6.** Association between 11 epigenetic fingerprint loci and medications. Associations were performed relative to both starting and stopping six classes of medications: diuretic, beta-blockers, anti-platelet, calcium channel blocker, statins, ACE-inhibitor, and angiotensin inhibitor. **Table S7.** Count of the genes within 1 Mb of each epigenetic fingerprint loci. **Table S8**. Integration of methylation, gene expression, and metabolomics for the suggestive (*P* < 0.001) gene expression-metabolite associations. **Figure S1.** Post hoc power estimations for the observed effects at our FDR cutoff of 0.15 for the initial screening EWAS. (ZIP 196 kb)

## Abbreviations

CVD: Cardiovascular disease
MI: Myocardial infarction
