# Supplementary material for: Analysis of repeated leukocyte DNA methylation assessments reveals persistent epigenetic alterations after an incident myocardial infarction
Source: Clin Epigenetics. 2018 Dec 27;10:161. doi: 10.1186/s13148-018-0588-7 (PMC6307146; doi:10.1186/s13148-018-0588-7)
Supplement: Supplementary file 1 — Supplemental methods, Table S1. CpGs from KORA EWAS. Those CpGs with a false discovery rate P < 0.15 in an epigenome-wide association study in KORA using the difference in methylation between the baseline and follow-up exams as the outcome (after adjustment for technical factors) and the occurrence of an MI as the predictor while adjusting for clinical covariates (at both baseline and follow-up) in a generalized estimating equations model. Table S2. The 174 CpGs which were retained from the initial elastic net model performed in KORA. Table S3. Epigenetic loci with non-zero coefficients from the NAS elastic net model. Table S4. AUC for the model fit with the loci with non-zero betas from the NAS elastic net in KORA, NAS, and InCHIANTI. Table S5. Medication usage in KORA at baseline and follow-up. We divide out the medication usage in KORA at (a) baseline and (b) follow-up for those individuals who did not develop and incident MI during the observation time (MI free) vs those that did (MI cases). Table S6. Association between 11 epigenetic fingerprint loci and medications. Associations were performed relative to both starting and stopping six classes of medications: diuretic, beta-blockers, anti-platelet, calcium channel blocker, statins, ACE-inhibitor, and angiotensin inhibitor. Table S7. Count of the genes within 1 Mb of each epigenetic fingerprint loci. Table S8. Integration of methylation, gene expression, and metabolomics for the suggestive (P < 0.001) gene expression-metabolite associations. Figure S1. Post hoc power estimations for the observed effects at our FDR cutoff of 0.15 for the initial screening EWAS. (ZIP 196 kb) [file 13148_2018_588_MOESM1_ESM.zip › Rev4 MI Fingerprint Supplemental Materials CE Submission v1.docx]

**Additional files**

**Supplemental Methods**

Data structure and equation for EWAS model:

The generalized estimationg equation model for the epigenome-wide association study is given below along with an example data structure for three individuals one of them (subject 2) had an incident MI. In the data structure the are indexed by subject "i" at time "t". The change in CpG (Δ CpG) was the independent variable and MI Occcurrence (M) was the predictor of interest. Thus the GEE model would be specified as:

Δ CpG_it_ = M_it_ + Confouders_it_ + ε_it_

Shown below is the data structure for such a model with confounders age and body mass index (BMI) shown for three subjects one of thow (subject 2) had an incident MI. C_i1_ = 0 as described in the Methods. The full model included the following confounders: age, sex, body mass index (BMI), type 2 diabetes, hypertension, physical activity, pack-years of smoking, and alcohol consumption (g/day).

| Subject ID | Time | Δ CpG | MI Occurrence | Age | BMI | … |
| --- | --- | --- | --- | --- | --- | --- |
| 1 | 1 | C_11_ | 0 | A_11_ | B_11_ |  |
| 1 | 2 | C_12_ | 0 | A_12_ | B_12_ |  |
| 2 | 1 | C_21_ | 0 | A_21_ | B_21_ |  |
| 2 | 2 | C_22_ | 1 | A_22_ | B_22_ |  |
| 3 | 1 | C_31_ | 0 | A_31_ | B_31_ |  |
| 3 | 2 | C_32_ | 0 | A_32_ | B_32_ |  |

**Cohort Descriptions (in alphabetical order)**

***Invecchiare nel Chianti (InCHIANTI)***

*Cohort Description*

The Invecchiare nel Chianti (InCHIANTI) Study is a population-based prospective cohort study of residents ages 20 or older from two areas in the Chianti region of Tuscany, Italy. Sampling and data collection procedures have been described elsewhere [[1](#_ENREF_1)]. Briefly, 1,326 participants donated a blood sample at baseline (1998-2000), of which 784 also donated a blood sample at the 9-year follow-up (2007-2009). DNA methylation was assayed using the Illumina Infinium HumanMethylation450 platform in DNA samples corresponding to participants with sufficient DNA at both baseline and Year 9 visits (n=499). All participants provided written informed consent to participate in this study. The study complied with the Declaration of Helsinki. The Italian National Institute of Research and Care on Aging Institutional Review Board approved the study protocol.

*MI assessment*

MI events were obtained from questionnaires with the following two questions: “Have you ever had an MI?” (baseline) and “Have you had an MI event since the last interview?” (follow-up). At follow-up interviews, participants are asked whether they have been told of a heart attack or hospitalized, and events are adjudicated according to specific algorithms that involve data from hospitalization, such as electrocardiographic findings and enzyme results. Prevalent cases of MI at baseline were excluded. Incident MI was then assessed over nine years of follow-up.

*DNA methylation data measurement*

Buffy coat genomic DNA was extracted using the Flex Star machine (Autogen, Inc., Holliston, MA, USA). DNA quantification and purity was tested using the Nanodrop1000 spectrophotometer (Thermo Scientific, Wilmington, DE, USA) prior to bisulfite conversion. Bisulfite conversion of the DNA was conducted using the EZ-96 DNA Methylation Kit (Zymo Research Corp., Irvine, CA), according to the manufacturer's protocol. DNA methylation quantification was conducting on the Illumina Infinium HumanMethylation450 BeadChip (Illumina Inc., San Diego, CA) as per the manufacturer’s protocol. Initial data analysis was performed using GenomeStudio 2011.1 (Model M Version 1.9.0, Illumina Inc.). Threshold call rate for inclusion of samples was 95%. Quality control of sample handling included comparison of clinically reported sex versus sex of the same samples determined by analysis of methylation levels of CpG sites on the X chromosome. Background correction and dye-bias equalization using the *noob* approach was implemented in the *minfi* R package [[2](#_ENREF_2), [3](#_ENREF_3)]. Between-array normalization performed separately for type I and II probes was conducted using the *dasen* approach, as implemented in the *wateRmelon* R package [[4](#_ENREF_4)].

*DNA methylation data preprocessing and quality control*

As part of the quality control process, samples were excluded if they represented extreme outliers on multidimensional scaling plots (>3 standard deviations). Genotyping data was also available in these samples from the Illumina HumanHap550-Quad+ Beadchip, described elsewhere [[5](#_ENREF_5)], as well as the Illumina Infinium HumanMethylation450 BeadChip. Among the 56 genotyping probes available on both arrays, we further excluded individual with blatant sample misidentification, defined as having more than 12 (21%) mismatched genotypes. These sample exclusions were further verified through beta value density plots, M versus U plots, and sex matching.

*Technical factor and cell count adjustment*

Cell proportions for B cells, granulocytes, monocytes, CD4+ T cells, CD8+ T cells, and natural killer cells were estimated from the DNA methylation data using the method described by Houseman et al. implemented in the *estimateCellCounts* function in the *minfi* R package [[3](#_ENREF_3), [6](#_ENREF_6)]. Models were adjusted for estimated cell counts, batch, slide, and array.

*Acknowledgements/Funding Sources*

The InCHIANTI study baseline (1998–2000) was supported as a “targeted project” (ICS110.1/RF97.71) by the Italian Ministry of Health and in part by the U.S. National Institute on Aging (Contracts 263 MD 9164 and 263 MD 821336). This work utilized the computational resources of the NIH HPC Biowulf cluster. (<http://hpc.nih.gov>). This research was supported in part by the Intramural Research Program of the NIH, National institute on Aging.

***Cooperative Health Research in the Region of Augsburg (KORA)***

*Cohort Description*

The Cooperative Health Research in the Region of Augsburg, Germany Survey 4 (KORA S4) is a population based survey of 4,261 individuals recruited from Augsburg, Germany from October, 1999 – April, 2001. All participants completed a detailed questionnaire which gathered information on medical history, clinical, and lifestyle factors. Peripheral blood samples were taken for later analyses [[7](#_ENREF_7)]. The KORA F4 survey is a seven-year follow-up study of the KORA S4 cohort conducted from 2006-2008, in which 3,080 KORA S4 participants were re-examined [[8](#_ENREF_8)].

For this analysis only individuals with quality controlled methylation data in KORA S4 and KORA F4 were used. We additionally restricted to individuals without a myocardial infarction at their baseline exam leaving 1103 participants for analysis. Of these individuals, 13 had an incident fatal or non-fatal myocardial infarction (MI) in the follow-up period between KORA S4 and KORA F4.

*MI Assessment*

Non-fatal MI events were reported to the Coronary Events Registry and linked to the cohorts using name and date of birth [[9](#_ENREF_9), [10](#_ENREF_10)]. For individuals who moved out of the study area, mailed questionnaires and general practitioner notes were used to validate MI. In the event that thequestionnaires were not returned the date of move was used as the loss to follow-up date. Fatal MI events found in population registries but not the Coronary Events Registry were classified using the general practitioner’s notes, hospital discharge letter, or ICD-9 code of the underlying cause of death.

*DNA methylation data measurement*

Genome-wide DNA methylation measurement at 485,577 genomic sites was performed using the Infinium HumanMethylation450K BeadChip® (Illumina, Inc., CA, USA)[[11](#_ENREF_11)] in 1814 KORA F4, and 1535 KORA S4 samples. The laboratory process has been described previously.[[12](#_ENREF_12)] Briefly, denaturated single-stranded genomic DNA was subjected to bisulfite treatment using the EZ-96 DNA Methylation Kit (Zymo Research, Orange, CA, USA). Bisulfite-converted samples were subjected to whole genome amplification, followed by enzymatic fragmentation and application to the BeadChips. The arrays were fluorescently stained and scanned with the Illumina HiScan SQ scanner. As readout, a methylated and an unmethylated signal count per CpG site are obtained. Counts were combined to β-values, defined as the ratio of the methylated signal intensity divided by the overall signal intensity - β-value = M/(M+U+α[[11](#_ENREF_11), [13](#_ENREF_13)] - with an offset (α) added as a regularization for the situation when both M and U are low, as recommended by Illumina [[13](#_ENREF_13)].

*DNA methylation data preprocessing and quality control*

DNA methylation data were preprocessed as follows: First, 65 probes that represent SNPs were excluded. Second, background correction was performed using the R package *minfi*, version 1.6.0 [[3](#_ENREF_3)]. Third, detection p-values were defined as the probability of a signal being detected above the background signal level, as estimated from negative control probes. Consequently, signals with detection p-values ≥ 0.01 were removed, since they indicate putatively unreliable signals. Similarly, signals summarized from less than three functional beads on the chip were characterized as potentially unreliable and removed from the data set. Observations with less than 95% CpG sites having a detection p-value > 0.01 (72 in KORA F4, 0 in KORA S4) were excluded.

To reduce the non-biological variability between observations, data were normalized using quantile normalization (QN) on the raw signal intensities[[14](#_ENREF_14)] . Precisely, QN was stratified to six probe categories based on probe type and color channel (i.e., Infinium I signals from beads targeting methylated CpG sites obtained through the red and the green color channels, Infinium I signals from beads targeting unmethylated CpG sites obtained through the red and the green color channels, and Infinium II signals obtained through the red and the green color channels) [[11](#_ENREF_11)] using the R package *limma*, version 3.16.5 [[15](#_ENREF_15)]. Furthermore, in order to correct the shift in the distribution of methylation values observed for the two different assay designs (Infinium I and Infinium II) present on the BeadChip, beta-mixture quantile normalization (BMIQ) was applied [[16](#_ENREF_16)] using the R package *watermelon*, version 1.0.3 [[4](#_ENREF_4)].

*Technical factor and cell count adjustment*

Technical factor adjusted methylation residuals were used for the analysis. To create these we adjusted each methylation probe β value for technical factors via the CPACOR method [[14](#_ENREF_14)] whereby 20 principal components derived from the control probes were included in the adjustment model. Cell counts as derived from the Housman estimates [[6](#_ENREF_6)] were also included. The following estimated cell counts were adjusted for in addition to the technical factors: granulocytes, monocytes, B cells, CD4+ T cells, CD8+ T cells and natural killer cells

*Acknowledgements/Funding Sources*

We would like to thank all KORA participants for their participation in the KORA cohort. This work was supported by the German Federal Ministry of Education and Research (BMBF) within the framework of the e:Med research and funding concept (e:AtheroSysMed, grant 01ZX1313A-2014). Part of this work received funding from the European Union Seventh Framework Programme under grant agreement [n°603288] (Systems Biology to Identify Molecular Targets for Vascular Disease Treatment – SysVasc; <http://www.sysvasc.eu/>).

***The Normative Aging Study (NAS)***

*Cohort Description*

The ongoing longitudinal US Department of Veterans Affairs (VA) Normative Aging Study (NAS) was established in 1963 and included men, 21-80 years old and free of known chronic medical conditions at entry [[17](#_ENREF_17)]. Subsequently participants were invited to medical examinations every three to five years. At each visit, men provided information on medical history, lifestyle, and demographic factors, and underwent physical examinations and laboratory tests. DNA samples were collected from 675 active participants between 1999-2007 [[17](#_ENREF_17)]. For the current analysis, participants were excluded if they had previously developed coronary heart disease at the ‘baseline’ examination 1(i.e. 1^st^ examination from which DNA methylation data was derived), if they did not have methylation data at both examination 1 and 2, or if they had non-MI forms of incident CHD developed between examination 1 and 2. The final sample size for analysis was 344. The NAS study was approved by the Institutional Review Boards (IRBs) of the participating institutions.

*MI ascertainment*

Experienced research staff coded cardiovascular outcomes using ICD-9 codes. Included MI cases were recognized MI (i.e. hospitalization with diagnostic ECG changes and/or biomarkers of MI)

*DNA Methylation measures*

DNA was extracted from buffy coat using the QIAamp DNA Blood Kit (QIAGEN, Valencia, CA). 500 ng of DNA was used to perform bisulfite conversion using the EZ-96 DNA Methylation Kit (Zymo Research, Orange, CA). To reduce the chip and plate effects, we used a two-stage age-stratified algorithm to randomise samples and ensure similar age distributions across chips and plates; 12 samples – which were sampled across all the age quartiles – were randomized to each chip, then chips were randomised to plates (each housing eight chips).

Quality control analysis was performed to remove samples and probes, where >1% of probes or samples, respectively, had a detection p-value > 0.05. The remaining samples were preprocessed using the Illumina-type background correction [[2](#_ENREF_2)] and normalized with the dye-bias and BMIQ adjustments [[18](#_ENREF_18)].

*NAS Technical covariates*

To avoid any batch effect of DNA methylation levels, we included technical covariates for plate, position of the chip, row and column as fixed effect in each analysis.

1. Ferrucci L, Bandinelli S, Benvenuti E, Di Iorio A, Macchi C, Harris TB, Guralnik JM: **Subsystems contributing to the decline in ability to walk: bridging the gap between epidemiology and geriatric practice in the InCHIANTI study.** *J Am Geriatr Soc* 2000, **48:**1618-1625.

2. Triche TJ, Jr., Weisenberger DJ, Van Den Berg D, Laird PW, Siegmund KD: **Low-level processing of Illumina Infinium DNA Methylation BeadArrays.** *Nucleic Acids Res* 2013, **41:**e90.

3. Aryee MJ, Jaffe AE, Corrada-Bravo H, Ladd-Acosta C, Feinberg AP, Hansen KD, Irizarry RA: **Minfi: a flexible and comprehensive Bioconductor package for the analysis of Infinium DNA methylation microarrays.** *Bioinformatics* 2014, **30:**1363-1369.

4. Pidsley R, CC YW, Volta M, Lunnon K, Mill J, Schalkwyk LC: **A data-driven approach to preprocessing Illumina 450K methylation array data.** *BMC Genomics* 2013, **14:**293.

5. Wood AR, Hernandez DG, Nalls MA, Yaghootkar H, Gibbs JR, Harries LW, Chong S, Moore M, Weedon MN, Guralnik JM, et al: **Allelic heterogeneity and more detailed analyses of known loci explain additional phenotypic variation and reveal complex patterns of association.** *Hum Mol Genet* 2011, **20:**4082-4092.

6. Houseman EA, Accomando WP, Koestler DC, Christensen BC, Marsit CJ, Nelson HH, Wiencke JK, Kelsey KT: **DNA methylation arrays as surrogate measures of cell mixture distribution.** *BMC Bioinformatics* 2012, **13:**86.

7. Holle R, Happich M, Lowel H, Wichmann HE: **KORA--a research platform for population based health research.** *Gesundheitswesen* 2005, **67 Suppl 1:**S19-25.

8. Rückert I-M, Heier M, Rathmann W, Baumeister SE, Döring A, Meisinger C: **Association between markers of fatty liver disease and impaired glucose regulation in men and women from the general population: the KORA-F4-study.** *PloS one* 2011, **6:**e22932.

9. Ziegler D, Rathmann W, Meisinger C, Dickhaus T, Mielck A: **Prevalence and risk factors of neuropathic pain in survivors of myocardial infarction with pre-diabetes and diabetes. The KORA Myocardial Infarction Registry.** *Eur J Pain* 2009, **13:**582-587.

10. Kirchberger I, Heier M, Kuch B, von Scheidt W, Meisinger C: **Presenting symptoms of myocardial infarction predict short- and long-term mortality: the MONICA/KORA Myocardial Infarction Registry.** *Am Heart J* 2012, **164:**856-861.

11. Bibikova M, Barnes B, Tsan C, Ho V, Klotzle B, Le JM, Delano D, Zhang L, Schroth GP, Gunderson KL, et al: **High density DNA methylation array with single CpG site resolution.** *Genomics* 2011, **98:**288-295.

12. Zeilinger S, Kühnel B, Klopp N, Baurecht H, Kleinschmidt A, Gieger C, Weidinger S, Lattka E, Adamski J, Peters A: **Tobacco smoking leads to extensive genome-wide changes in DNA methylation.** *PloS one* 2013, **8:**e63812.

13. Du P, Zhang X, Huang CC, Jafari N, Kibbe WA, Hou L, Lin SM: **Comparison of Beta-value and M-value methods for quantifying methylation levels by microarray analysis.** *BMC Bioinformatics* 2010, **11:**587.

14. Lehne B, Drong AW, Loh M, Zhang W, Scott WR, Tan S-T, Afzal U, Scott J, Jarvelin M-R, Elliott P: **A coherent approach for analysis of the Illumina HumanMethylation450 BeadChip improves data quality and performance in epigenome-wide association studies.** *Genome biology* 2015, **16:**1.

15. Smyth GK: **Limma: linear models for microarray data.** In *Bioinformatics and computational biology solutions using R and Bioconductor.* Springer; 2005: 397-420

16. Teschendorff AE, Marabita F, Lechner M, Bartlett T, Tegner J, Gomez-Cabrero D, Beck S: **A beta-mixture quantile normalization method for correcting probe design bias in Illumina Infinium 450 k DNA methylation data.** *Bioinformatics* 2013, **29:**189-196.

17. Bell B, Rose CL, Damon A: **The Veterans Administration longitudinal study of healthy aging.** *Gerontologist* 1966, **6:**179-184.

18. Teschendorff AE, Marabita F, Lechner M, Bartlett T, Tegner J, Gomez-Cabrero D, Beck S: **A beta-mixture quantile normalization method for correcting probe design bias in Illumina Infinium 450 k DNA methylation data.** *Bioinformatics* 2013, **29:**189-196.

**Tables**

**Additional files Tables 1, 2, and 6 can be found as separate additional data tables. They are not copied into this document due to their large size.**

**Table S1: CpGs from KORA EWAS.** Those CpGs with a false discovery rate P < 0.15 in an epigenome-wide association study in KORA using the difference in methylation between the baseline and follow-up exams as the outcome (after adjustment for technical factors) and the occurrence of an MI as the predictor while adjusting for clinical covariates (at both baseline and follow-up) in a generalized estimating equations model. Cases were those individuals who had an incident MI between the baseline and follow-up examinations. Controls were those individuals who did not have an incident MI between baseline and follow-up examinations. Complete model estimates for each CpG available upon request. Avg = average; Adj Methylation = methylation beta-values adjusted for cell counts and technical factors (20 principal components from control probes); BP = base-pair location; CHR = chromosome; FDR = false-discovery rate; Gene = nearest gene; LCI = lower 95% confidence interval; UCI = upper 95% confidence interval.

**Table S2:** The 174 CpGs which were retained from the initial elastic net model performed in KORA. After refinement in NAS, 11 of these CpGs were retained in the epigenetic fingerprint. BP (Mb) = base-pair location in mega-bases; Chrom = chromosome; CpG = DNA methylation probe name; Gene = nearest gene

**Table S3:** Epigenetic loci with non-zero coefficients from the NAS elastic net model, CHR=chromosome; BP= location given in megabases, DHS= DNase I hypersensitive site

| CpG site | CHR | BP (Mb) | Annotated Gene | Relation to Island (UCSC) |
| --- | --- | --- | --- | --- |
| cg00699486 | 6 | 166.00 |  |  |
| cg02628823 | 4 | 141.00 |  | South Shore |
| cg03458344 | 1 | 171.00 | C1orf129 |  |
| cg07311024 | 12 | 75.79 | *GLIPR1L2* | Island |
| cg08193363 | 13 | 32.61 | *FRY* | North Shore |
| cg10073091 | 1 | 55.35 | *DHCR24* | North Shore |
| cg11955541 | 1 | 145.00 | *PDE4DIP* | Island |
| cg19569340 | 7 | 5.82 | *RNF216* |  |
| cg21609024 | 1 | 53.80 | *LRP8* |  |
| cg23074119 | 14 | 78.17 | *ALKBH1* |  |
| cg23541257 | 19 | 18.10 | *KCNN1* | South Shelf |

**Table S4:** AUC for the model fit with the loci with non-zero betas from the NAS elastic net in KORA, NAS and InCHIANTI

|  | N | N MI | AUC | P (AUC) |
| --- | --- | --- | --- | --- |
| KORA | 1103 | 13 | 0.97 | 2.2E-09 |
| NAS | 344 | 14 | 0.91 | 8.1E-08 |
| InCHIANTI | 443 | 50 | 0.65 | 7.6E-03 |

**Table S5:** Medication usage in KORA at baseline and follow-up. We divide out the medication usage in KORA at (a) baseline and (b) follow-up for those individuals who did not develop and incident MI during the observation time (MI free) vs those that did (MI cases).

| a. | MI free (Baseline) | MI cases (Baseline) |
| --- | --- | --- |
| Beta-blockers | 135 (12.4%) | 4 (30.8%) |
| ACE-inhibitor | 73 (6.71%) | 2 (15.4%) |
| Diuretic | 89 (8.18%) | 2 (15.4%) |
| Angiotensin receptor blocker | 22 (2.02%) | 1 (7.69%) |
| Calcium channel blocker | 50 (4.6%) | 1 (7.69%) |
| Statins | 69 (6.34%) | 4 (30.8%) |
| Anti-platlet | 71 (6.53%) | 6 (46.2%) |
|  |  |  |
| b. | MI free (Follow-up) | MI cases (Follow-up) |
| Beta-blockers | 242 (22.2%) | 13 (100%) |
| ACE-inhibitor | 187 (17.2%) | 6 (46.2%) |
| Diuretic | 227 (20.8%) | 9 (69.2%) |
| Angiotensin receptor blocker | 98 (9%) | 2 (15.4%) |
| Calcium channel blocker | 96 (8.82%) | 1 (7.69%) |
| Statins | 161 (14.8%) | 12 (92.3%) |
| Anti-platlet | 132 (12.1%) | 12 (92.3%) |

**Table S6:** Association between 11 epigenetic fingerprint loci and medications. Associations were performed relative to both starting and stopping six classes of medications: diuretic, beta-blockers, anti-platelet, calcium channel blocker, statins, ACE-inhibitor, and angiotensin inhibitor. Beta = effect estimate; CpG = DNA methylation probe name; SE = standard error

**Table S7:** Count of the genes within 1Mb of each epigenetic fingerprint loci

| Fingerprint locus | # genes within 1Mb |
| --- | --- |
| cg00699486 | 14 |
| cg03458344 | 24 |
| cg07311024 | 17 |
| cg08193363 | 16 |
| cg10073091 | 31 |
| cg11955541 | 59 |
| cg21609024 | 45 |
| cg23074119 | 33 |
| cg23541257 | 87 |

**Table S8:** Integration of methylation, gene expression, and metabolomics for the suggestive (P < 0.001) gene-expression metabolite associations. FDR significant associations are given in bold. CpG = methylation locus; eQTL = analyses of genetic variants in association with cis-gene expression; eQTMetab FDR P = FDR P-value for eQTMetab Spearman correlation; eQTMetab P = P-value for eQTMetab Spearman correlation; eQTMetab Spearman Correlation = Spearman correlation between gene expression metabolite concentration; FDR = false discovery rate (Benjamini-Hochberg); GEX = gene expression; Metab = metabolite * = Bonferroni significant P-value

| CpG | Gene expression Probe | Anntated gene | eQTL Beta | eQTL P | Metabolite | eQTMetab Spearman Correlation | eQTMetab P | eQTMetab FDR P |
| --- | --- | --- | --- | --- | --- | --- | --- | --- |
| **cg23541257** | **ILMN_1797005** | **PGLS** | **-1.36** | **0.03** | **lactate** | **-0.18** | **2.40E-06*** | **0.008** |
| **cg21609024** | **ILMN_1671568** | **ECHDC2** | **2.02** | **0.03** | **mannose** | **-0.16** | **1.70E-05*** | **0.017** |
| **cg10073091** | **ILMN_1681340** | **HSPB11** | **-4.29** | **0.01** | **Glycerol(18:2(9Z,12Z)/0:0/0:0)** | **0.16** | **1.80E-05** | **0.017** |
| **cg10073091** | **ILMN_1681340** | **HSPB11** | **-4.29** | **0.01** | **4-hydroxyphenyllactate** | **0.16** | **1.90E-05** | **0.017** |
| **cg10073091** | **ILMN_1681340** | **HSPB11** | **-4.29** | **0.01** | **lactate** | **0.16** | **2.60E-05** | **0.018** |
| **cg10073091** | **ILMN_1681340** | **HSPB11** | **-4.29** | **0.01** | **proline** | **0.15** | **3.90E-05** | **0.021** |
| **cg23541257** | **ILMN_1797005** | **PGLS** | **-1.36** | **0.03** | **hippurate** | **0.15** | **4.20E-05** | **0.021** |
| **cg10073091** | **ILMN_1681340** | **HSPB11** | **-4.29** | **0.01** | **inosine** | **-0.15** | **1.30E-04** | **0.038** |
| **cg10073091** | **ILMN_1681340** | **HSPB11** | **-4.29** | **0.01** | **inositol 1-phosphate** | **0.16** | **1.20E-04** | **0.038** |
| **cg23541257** | **ILMN_1797005** | **PGLS** | **-1.36** | **0.03** | **3-methyl-2-oxobutyrate** | **-0.15** | **1.00E-04** | **0.038** |
| **cg23541257** | **ILMN_1797005** | **PGLS** | **-1.36** | **0.03** | **3-methyl-2-oxovalerate** | **-0.15** | **8.80E-05** | **0.038** |
| **cg23541257** | **ILMN_1797005** | **PGLS** | **-1.36** | **0.03** | **3-phenylpropionatehydrocinnamate** | **0.17** | **1.30E-04** | **0.038** |
| cg10073091 | ILMN_1681340 | HSPB11 | -4.29 | 0.01 | Glycerol(16:0/0:0/0:0) | 0.14 | 3.00E-04 | 0.079 |
| cg23541257 | ILMN_1654571 | FCHO1 | -1.52 | 0.02 | S-glutathionyl-L-cysteine | 0.14 | 4.20E-04 | 0.105 |
| cg23541257 | ILMN_1654571 | FCHO1 | -1.52 | 0.02 | hippurate | 0.13 | 5.20E-04 | 0.107 |
| cg10073091 | ILMN_1681340 | HSPB11 | -4.29 | 0.01 | Glycerol(18:1(9Z)/0:0/0:0) | 0.13 | 5.20E-04 | 0.107 |
| cg23541257 | ILMN_1742917 | NXNL1 | 1 | 0.03 | 7-methylguanine | -0.13 | 4.70E-04 | 0.107 |
| cg21609024 | ILMN_1671568 | ECHDC2 | 2.02 | 0.03 | caprate (10:0) | 0.13 | 6.50E-04 | 0.121 |
| cg23541257 | ILMN_1654571 | FCHO1 | -1.52 | 0.02 | 7-hcoa | -0.13 | 7.30E-04 | 0.121 |
| cg23541257 | ILMN_1797005 | PGLS | -1.36 | 0.03 | alphahydroxyisovalerate | -0.13 | 7.10E-04 | 0.121 |
| cg19569340 | ILMN_1652445 | RAC1 | 0.31 | 0.02 | guanosine | 0.13 | 7.10E-04 | 0.121 |
| cg10073091 | ILMN_1673544 | TCEANC2 | 2.37 | 0.00 | tyrosine | -0.12 | 9.30E-04 | 0.148 |


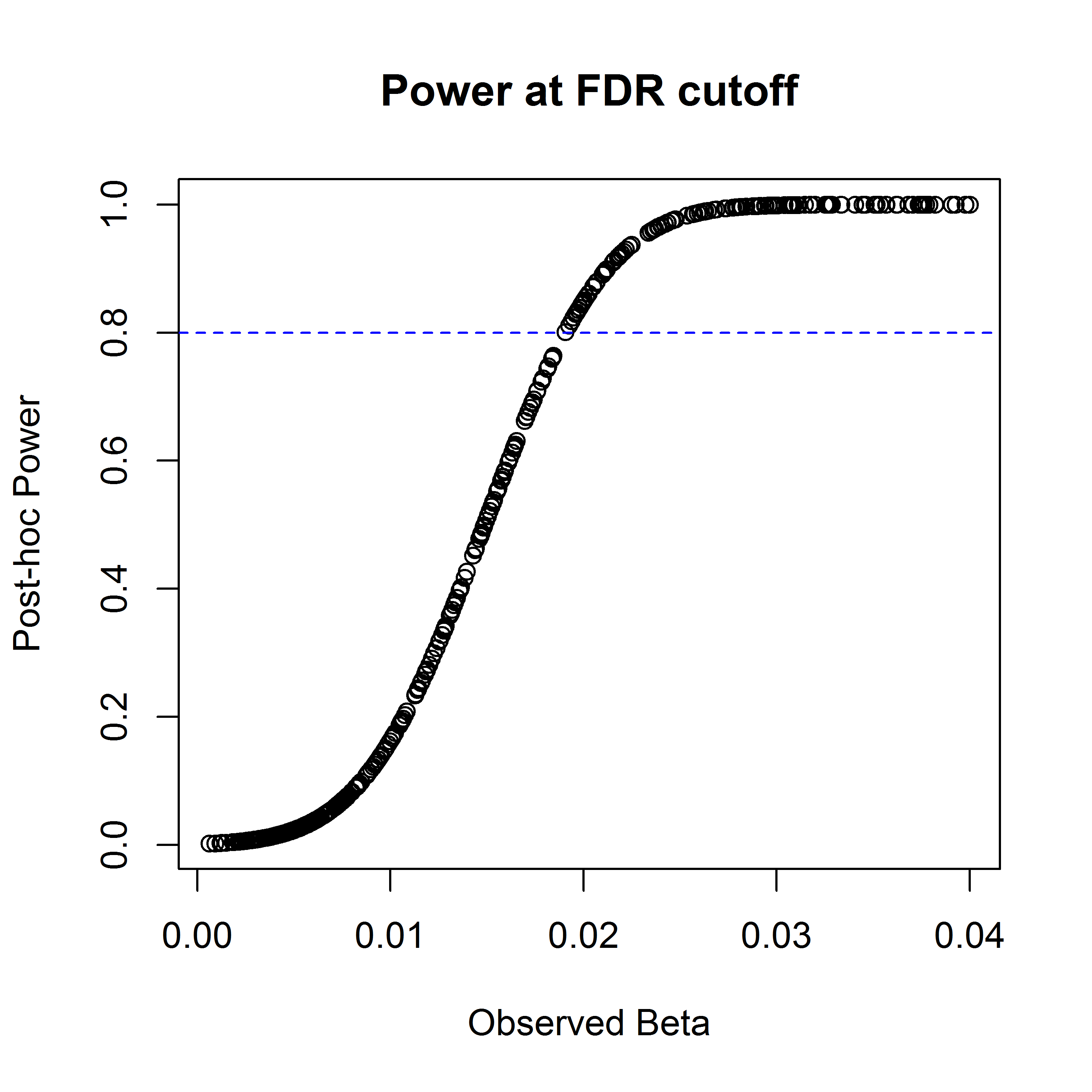


**Figure S1**: Post-hoc power estimations for the observed effects at our FDR cutoff of 0.15 for the initial screening EWAS. An FDR cutoff of 0.15 for the initial screening EWAS corresponded to a p-value cutoff of 0.0027. As expected given the small numbers only a few of the loci would be expected 80% power threshold (blue dashed line). Of the 435 FDR significant loci only 152 had betas which would have yielded 80% power according to our estimations. Power calculations were done using the longpower package in R and accounted for the difference in the numbers of those who had an MI during follow-up versus those who did not as well as the standard deviation of the CpGs for those who had an MI versus those who did not. For estimation purposes the median of the standard deviation and correlation between CpGs between baseline and follow-up was used.
